# Supplementary material for: Bioconjugation of Carbohydrates to Gelatin Sponges Promoting 3D Cell Cultures
Source: Biomimetics (Basel). 2023 May 6;8(2):193. doi: 10.3390/biomimetics8020193 (PMC10204423; doi:10.3390/biomimetics8020193)
Supplement: Supplementary file 1 [file biomimetics-08-00193-s001.zip › biomimetics-2354586-supplementary.pdf]

Supplementary Information

Bioconjugation of Carbohydrates to Gelatin Sponges Promoting 3D Cell Cultures

Antonietta Pepe [1,*], Antonio Laezza [1], Angela Ostuni [2], Alessandra Scelsi [1], Alessandro Laurita [3] and Brigida Bochicchio [1,*]

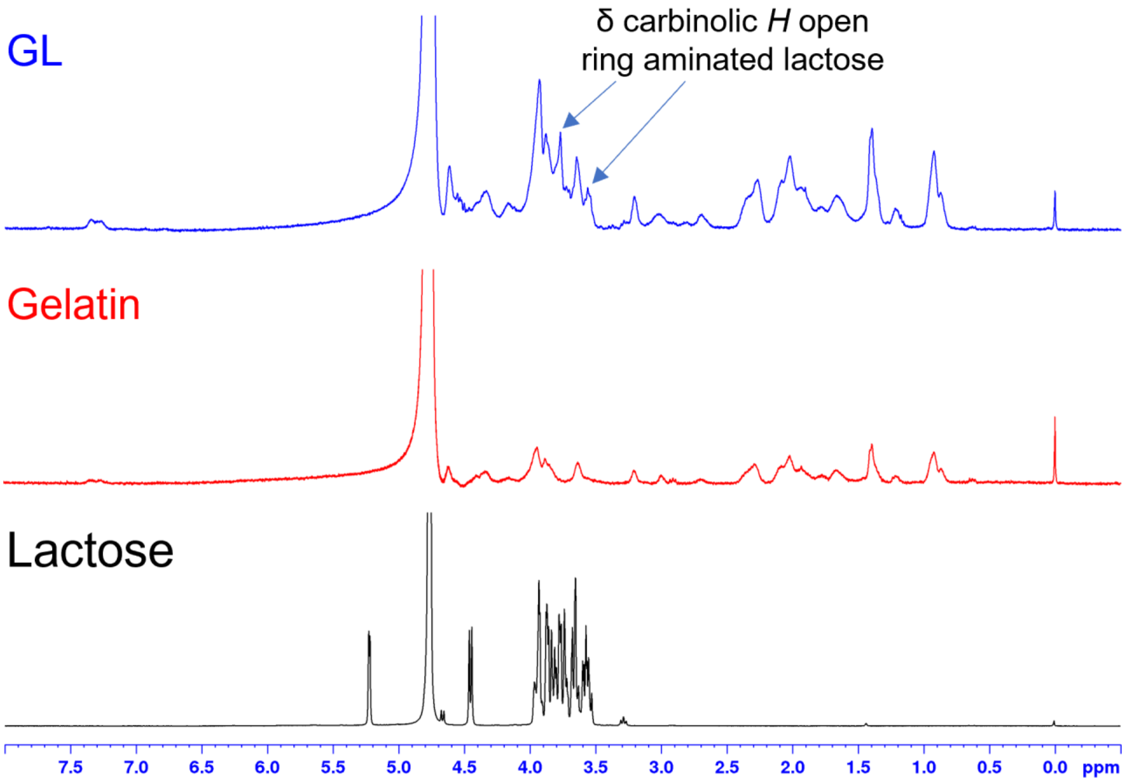

Figure S1. Characterization of gelatin derivatives by ^{1}H NMR spectra recorded in D_2O at 289 K. ^{1}H NMR spectra of lactose (black curve), gelatin (red curve) and GL (blue curve).

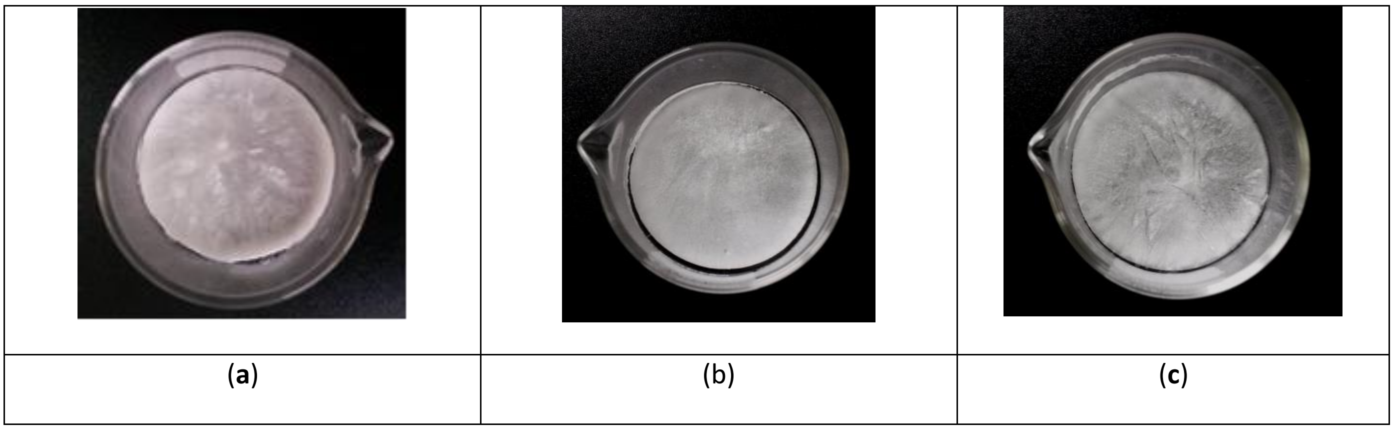

(a)	(b)	(c)

Figure S2. Images of the GS (a), GS-M (b) and GS-L (c) sponges after freeze-drying.

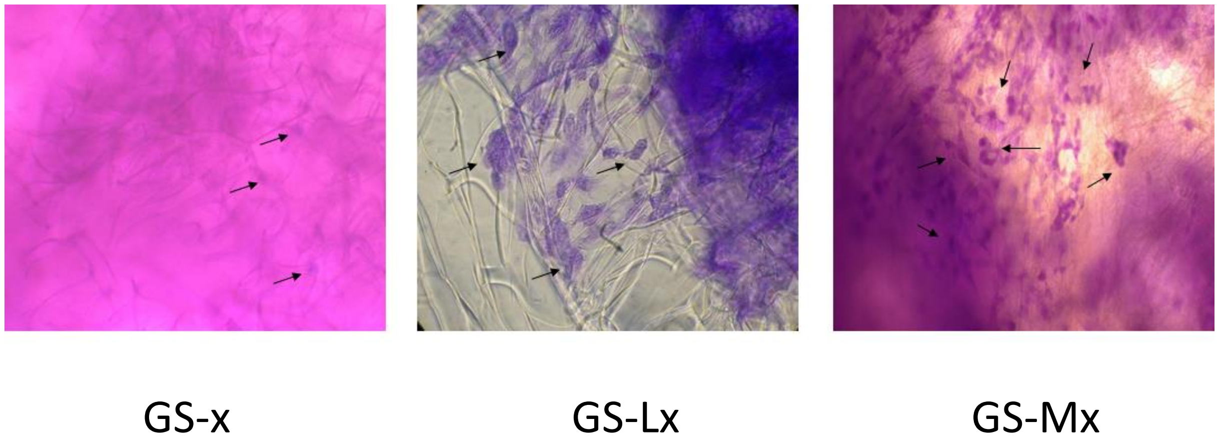

Figure S3. Microscope images of HepG2 cells grown on GS-x, GS-Lx, and GS-Mx sponges for 13 days, stained with crystal violet. Arrows indicate cells.
